# Erythropoietin improves long-term neurological outcome in acute ischemic stroke patients: a randomized, prospective, placebo-controlled clinical trial

**DOI:** 10.1186/s13054-015-0761-8

**Published:** 2015-02-25

**Authors:** Tzu-Hsien Tsai, Cheng-Hsien Lu, Christopher Glenn Wallace, Wen-Neng Chang, Shu-Feng Chen, Chi-Ren Huang, Nai-Wen Tsai, Min-Yu Lan, Pei-Hsun Sung, Chu-Feng Liu, Hon-Kan Yip

**Affiliations:** Division of Cardiology, Department of Internal Medicine, Kaohsiung Chang Gung Memorial Hospital, Kaohsiung 123, Ta Pei Road, Niao Sung Hsiang, Kaohsiung, Hsien 83301 Taiwan; Department of Neurology, Kaohsiung Chang Gung Memorial Hospital, Kaohsiung 123, Ta Pei Road, Niao Sung Hsiang, Kaohsiung, Hsien 83301 Taiwan; Department of Plastic Surgery, University Hospital of South Manchester, Southmoor Road, Manchester, M23 9LT UK; Department of Emergency Medicine, Kaohsiung Chang Gung Memorial Hospital, Kaohsiung 123, Ta Pei Road, Niao Sung Hsiang, Kaohsiung, Hsien 83301 Taiwan; Center for Translational Research in Biomedical Sciences, Kaohsiung Chang Gung Memorial Hospital, Kaohsiung 123, Ta Pei Road, Niao Sung Hsiang, Kaohsiung, Hsien 83301 Taiwan; Institute of Shock Wave Medicine and Tissue Engineering, Kaohsiung Chang Gung Memorial Hospital, 123, Ta Pei Road, Niao Sung Hsiang, Kaohsiung, Hsien 83301 Taiwan; Chang Gung University College of Medicine, 123, Ta Pei Road, Niao Sung Hsiang, Kaohsiung, Hsien 83301 Taiwan

## Abstract

**Introduction:**

Mortality and disability following ischemic stroke (IS) remains unacceptably high with respect to the conventional therapies. This study tested the effect of erythropoietin (EPO) on long-term neurological outcome in patients after acute IS. This study aimed to evaluate the safety and efficacy of two consecutive doses of EPO (5,000 IU/dose, subcutaneously administered at 48 hours and 72 hours after acute IS) on improving the 90-day combined endpoint of recurrent stroke or death that has been previously reported. A secondary objective was to evaluate the long-term (that is, five years) outcome of patients who received EPO.

**Methods:**

This was a prospective, randomized, placebo-controlled trial that was conducted between October 2008 and March 2010 in a tertiary referral center. IS stroke patients who were eligible for EPO therapy were enrolled into the study.

**Results:**

The results showed that long-term recurrent stroke and mortality did not differ between group 1 (placebo-control; n = 71) and group 2 (EPO-treated; n = 71).

Long-term Barthel index of <35 (defining a severe neurological deficit) was lower in group 2 than group 1 (*P* = 0.007). Multiple-stepwise logistic-regression analysis showed that EPO therapy was significantly and independently predictive of freedom from a Barthel index of <35 (*P* = 0.029). Long-term major adverse neurological event (MANE; defined as: death, recurrent stroke, or long-term Barthel index < 35) was lower in group 2 than group 1 (*P* = 0.04). Log-Rank test showed that MANE-free rate was higher in group 2 than group 1 (*P* = 0.031). Multiple-stepwise Cox-regression analysis showed that EPO therapy and higher Barthel Index at day 90 were independently predictive of freedom from long-term MANE (all *P* <0.04).

**Conclusion:**

EPO therapy significantly improved long-term neurological outcomes in patients after IS.

**Trial registration:**

ISRCTN71371114. Registered 10 October 2008.

## Introduction

Acute ischemic stroke (IS) accounts for greater than 70% of all types of acute stroke and is a leading cause of death, disability, and dependence worldwide. Despite new diagnostic tools [1,2] and the refinements of new anti-platelet agents [3,4], the morbidity, mortality, and residual severe disability following IS have remained unacceptably high over decades with respect to those of conventional therapies [5,6]. Most patients with disabilities from IS remain dependent on others and usually have unfavorable long-term outcomes [7,8]. Evidence is growing that thrombolytic therapy with tissue plasminogen activator (tPA) may significantly improve patients’ clinical outcome after acute IS; however, not all acute IS patients fit the criteria for tPA therapy [9-11]. A new, safe, and efficacious treatment option needs to be developed for those patients with acute IS who are not candidates for tPA therapy.

Erythropoietin (EPO) was first utilized for treating anemic patients of various etiologies, such as patients with end-stage renal disease on regular hemodialysis [12,13]. Intriguingly, EPO also appears to have pleiotropic effects, such as anti-ischemic and anti-apoptotic properties [14-16], promotion of neovascularization, mobilization of endothelial progenitor cells (EPCs), and enhancement of angiogenesis [17-19]. EPO has previously been prescribed to acute IS patients in some clinical studies, but the neuroprotective effect of EPO is poorly documented and results have been inconsistent [20-22]. Given the pleiotropic effects of EPO therapy, the inconsistent clinical outcomes of EPO therapy after acute IS in clinical reports and our previous finding that an increase in circulating levels of EPCs in patients after acute IS was significantly associated with favorable clinical outcomes [23], we performed a prospective, randomized, and placebo-controlled trial [24]. The primary objective of this clinical trial was to evaluate the safety and efficacy of two consecutive doses of EPO (5,000 IU per dose, subcutaneously administered at 48 hours and 72 hours after acute IS, Epoetin beta; Roche) on improving the 90-day combined endpoint of recurrent stroke or death [24]. The secondary objectives of this study were: to establish the time course of circulating levels of EPCs in patients after acute IS; to investigate the ability of two doses of EPO to enhance circulating EPC levels; and to assess 5-year outcomes of patients who received EPO therapy after acute IS. We report, herein, the findings of the 5-year outcomes of this clinical trial.

The two doses of EPO administration to the acute IS patients were basic in consideration of safety and the clinical practice of EPO use for hemodialysis patients each week. Additionally, the chosen time point of EPO treatment at 48 hours and 72 hours after acute IS was owing to the fact that time was required for magnetic resonance imaging (MRI) study and enrollment as well as the delay in presentation to hospital for most acute IS patients.

## Materials and methods

### Study protocol and calculation of sample size for the specific objective

This clinical trial was approved by the Institutional Review Committee on Human Research in Chang Gung Memorial Hospital (No 96-1381A) in 2007 and was conducted at Kaohsiung Chang Gung Memorial Hospital. Informed consent was obtained from all participating patients or their legal representatives. Funding for this study was delivered by a program grant from the National Science Council, Taiwan, Republic of China (NSC-97-2314-B-182A-090-MY2).

This was a prospective, randomized, placebo-controlled trial. The study included consecutively admitted acute IS patients who were not candidates for tPA therapy at a single facility between October 2008 and March 2010. The study design protocol, definitions and exclusion criteria, calculation of sample size for specific objectives, neurological assessment methods, blood sampling and assessment of circulating EPC levels, and medications have been extensively detailed in our previous report [24], and were described because the study included consecutively admitted acute IS patients at a single facility between October 2008 and March 2010. For the primary objective of the study, an estimated sample size of 106 study patients in each group was based on the effective size with α = 0.05, a power of 80%, and anticipation of a combined end point of 14.0% in placebo control versus 4.0% with EPO therapy. For the secondary objective of this study, an estimate sample size of 93 study patients in each group was based on the effective size with α = 0.05, a power of 80%, an average difference in the circulating level of EPCs between the EPO therapy and placebo-control groups of 0.32%, and a standard deviation of circulating level of EPCs in EPO therapy of 0.7%. A 20% rate of protocol violations and incomplete follow-up were assumed. Calculation of the sample size for the specific objective was based on our recent report [23].

### Definition of ischemic stroke, and inclusion and exclusion criteria

The definition of acute IS and the exclusion criteria have been described in our previous report [24]. In detail, stroke was defined as sudden onset of loss of global or focal cerebral function persisting for more than 24 hours. Patients of any age with acute IS were eligible. Inclusion criteria included a score >2 on the National Institutes of Health Stroke Scale (NIHSS; scores up to 8 indicate moderate neurological status disability) and a time window ≤48 hours from onset of symptoms to blood sampling (at 48 hours after IS) and study drug administration (time to treatment just after blood sampling). Patients with history of the following were excluded from the study: intracranial hemorrhage, surgery or trauma within the preceding 3 months, abnormal liver function, hematology disorders, renal insufficiency (serum creatinine >1.5 mg/dl), malignancy, febrile disorders, acute or chronic inflammatory disease at study entry, liver cirrhosis, atrial fibrillation, congestive heart failure, contraindications for MRI examination, no evidence of acute IS by MRI study, myeloproliferative disorder, antibodies or being allergic to EPO, pregnancy, tPA therapy for acute IS, or a hemoglobin level >15.0 gm/dl.

### Neurological assessment

Evaluation of the physical function and degree of neurological impairment in the stroke patients was based on the NIHSS [25] during the acute (48 hours), convalescent (day 21), and chronic (day 90) phases of stroke by neurologists blinded to the treatment allocation (double-blind study). Moderate neurological impairment (that is, neurological sequelae that require partial support in daily activities) was defined as a score ≥8 on the NIHSS, a modified criterion reported previously [26]. In addition to the NIHSS, assessments only during admission included functional measures.

The Barthel Index [27], which ranges from 100 (no deficit) to 0 (complete dependence or death), was utilized for long-term functional assessment as evaluated by neurologists blinded to the treatment allocation (double-blind study). Barthel Index <35 was defined as a severe disability (life dependence) [28-31]. A long-term major adverse neurological event (MANE) was defined as recurrent stroke, Barthel Index <35, or long-term mortality (defined as death observed >90 days after acute IS). All of these variables were meticulously documented prospectively.

### Brain computerized tomography and MRI definition of acute ischemic stroke

The radiological diagnosis of acute IS included brain computed tomography; that is, a new finding of low attenuation density in the focal or diffuse brain area. Additionally, the MRI findings of acute IS were defined as follows: a typical acute IS (24 hours to 1 week) appears hyperintense on T2-weighted, diffusion-weighted, and fluid-attenuated inversion recovery images; and appears hypointense on T1-weighted image and apparent diffusion coefficient mapping.

### Protocol for 5-year follow-up

All patients who survived acute IS were discharged from hospital. Self-caring patients returned home. Dependent patients with severe disability were transferred to nursing and rehabilitation centers for special care, and were regularly followed-up in outpatients and/or by telephone.

### Number of patients eligible for long-term follow-up

In this clinical trial, 84 patients received placebo therapy. One patient died during hospitalization, leaving 83 patients who were followed-up in the outpatient department for 90 days. Of these, six patients declined involvement in the long-term follow-up study and were therefore withdrawn from the study after day-90 IS. An additional six patients were lost to follow up after day-90 IS. The remaining 71 patients served as group 1 (placebo control) of this long-term follow-up study.

Another 83 patients received EPO therapy. Two patients died during hospitalization, leaving 81 patients who were followed-up in the outpatient department for 90 days. Of these, five patients declined involvement in the long-term follow-up study and were therefore withdrawn from the study after day-90 IS. An additional five patients were lost to follow up after day-90 IS. The remaining 71 patients served as group 2 (EPO therapy) of this long-term follow-up study.

### Definitions

Barthel Index <35 was defined as a severe neurological deficit; this definition was modified from previous reports [28,31]. Major adverse cardiac event was defined as myocardial infarction or cardiovascular-related death. MANE was defined as recurrent stroke after day-90 IS, a long-term Barthel Index <35, or any cause of long-term death (that is, death after day-90 IS).

### Medications for outpatients

Aspirin was the first-choice medication for all IS patients unless they were allergic or intolerant to it, including a history of peptic ulcer or upper gastrointestinal tract bleeding during aspirin therapy. Clopidogrel was used in patients intolerant to aspirin. Other commonly used drugs included statins, oral hypoglycemics, angiotensin-converting enzyme inhibitors/angiotensin II type I receptor blockers, diuretics, calcium channel blocking agents, and beta-blockers.

### Statistical analyses

Data were expressed as means ± standard deviation or percentage of patients where appropriate. For the clinical and laboratory variables, comparisons were carried out using an independent *t* test for continuous variables and Fisher’s exact test or a chi-square test for categorical variables. A log-rank test was used to determine the MANE free rate and multiple stepwise Cox regression analysis was used to predict freedom from long-term MANE. All analyses were conducted using SAS statistical software for Windows version 8.2 (SAS Institute, Cary, NC, USA). *P* <0.05 was considered statistically significant.

## Results

### Comparison of baseline characteristics of patients with and without erythropoietin treatment

Table 1 presents the baseline variables of acute IS patients in both groups. There were no significant differences in terms of age, gender and incidence of coronary artery disease risk factors. Additionally, the incidences of previous stroke by history or brain MRI, previous myocardial infarction, or significant coronary artery disease did not differ between groups 1 and 2.

**Table 1:** **Comparison of baseline characteristics between patients treated with and without erythropoietin at admission**

**Variable**	**Group 1 (** ***n*** **= 71)**	**Group 2 (** ***n*** **= 71)**	***P*** **value** ^**a**^
Age (years)	65.6 ± 11.3	64.1 ± 11.4	0.481
Male	64.8% (46)	69.0% (49)	0.593
Hypertension	71.8% (51)	63.4% (45)	0.282
Diabetes mellitus	31% (22)	36.6% (26)	0.478
Current smoking	25.4% (18)	33.8% (24)	0.270
Previous stroke by history	19.7% (14)	23.9% (17)	0.542
Previous stroke by brain MRI	56.3% (40)	63.4% (45)	0.392
Obstructive CAD^b^	12.7% (9)	19.7% (14)	0.255
Old myocardial infarction	7.0% (5)	9.9% (7)	0.546
RBC count (× 10^6^/ml)	4.68 ± 0.69	4.72 ± 0.69	0.743
Hemoglobin (g/dl)	14.0 ± 1.9	14.1 ± 1.8	0.876
WBC count (× 10^3^/ml)	7.72 ± 2.35	7.81 ± 2.48	0.821
Total cholesterol level	189.1 ± 40.1	190.2 ± 36.4	0.864
HDL (mg/dl)	50.3 ± 18.1	45.6 ± 10.9	0.062
LDL (mg/dl)	114.0 ± 35.4	118.8 ± 31.9	0.415
Creatinine (mg/dl)	1.02 ± 0.41	1.01 ± 0.38	0.916
BMI (kg/m^2^)	23.9 ± 3.8	24.9 ± 3.3	0.086
HbA1C	6.88 ± 1.83	6.75 ± 1.92	0.699
SBP (mmHg)	143 ± 21	145 ± 23	0.669
DBP (mmHg)	80 ± 29	83 ± 12	0.098
Carotid artery stenosis^c^	15.3% (11)	23.9% (17)	0.192
Statin therapy	45.8% (33)	43.7% (31)	0.794
ACEI/ARB therapy	34.7% (25)	38% (27)	0.681

Data are expressed as mean ± standard deviation or percentage (number) of patients. Group 1, without erythropoietin treatment; Group 2, with erythropoietin treatment. ACEI/ARB, angiotensin-converting enzyme inhibitor/angiotensin II type I receptor blocker; BMI, body mass index; CAD, coronary artery disease (defined as >50% stenosis of epicardial vessel by angiographic finding); DBP, diastolic blood pressure; HbA1C, hemoglobin A1C; HDL, high-density lipoprotein; LDL, low-density lipoprotein; MRI, magnetic resonance imaging; RBC, red blood cell; SBP, systolic blood pressure; WBC, white blood cell. ^a^By *t* test or chi-square test. ^b^Defined as the coronary obstruction >50% at least one epicardial vessel. ^c^Defined as common carotid artery or internal carotid artery stenosis >50%.

The laboratory findings showed that the red blood cell count, the white blood cell count, and the levels of hemoglobin, total cholesterol, high-density lipoprotein, low-density lipoprotein and creatinine were similar between groups 1 and 2. Systolic and diastolic blood pressures, body mass index and the incidences of significant carotid artery stenosis, and use of statins or angiotensin converting enzyme inhibitor/angiotensin II type I receptor blockers did not differ significantly between groups 1 and 2.

### Time courses of circulating levels of EPCs and hematological findings between groups 1 and 2

In this clinical trial, blood samples were obtained at 48 hours (acute phase) and on day 7 (recovery phase) and day 21 (convalescent phase) after acute IS to determine the time courses of circulating levels of EPCs (CD31/CD34^+^ cells and KDR/CD34^+^ cells) in IS patients (see Table 2). The hematological components of patients at day 21 were also assessed to determine any possible impact of EPO therapy on them. The results showed that the circulating levels of EPCs at 48 hours and on day 7 after acute IS did not differ between these two groups. However, by day 21 after IS, the circulating levels of EPCs were significantly higher in group 2 than in group 1. Conversely, the red blood cell and white blood cell counts and the levels of hematocrit and hemoglobin did not differ between groups 1 and 2. These findings implicate that this regimen of EPO therapy slowly enhanced the circulating levels of EPCs but did not significantly affect the hematological components.

**Table 2:** **Time courses of circulating level of EPCs and hematological findings between the two groups of patients**

**Variable**	**Group 1 (** ***n*** **= 71)**	**Group 2 (** ***n*** **= 71)**	***P*** **value**
Circulating level of EPCs at 48 hours			
CD31/CD34 (%)	1.69 ± 1.01	1.59 ± 0.91	0.514
KDR/CD34 (%)	1.34 ± 0.92	1.31 ± 0.76	0.823
Circulating level of EPCs at day 7			
CD31/CD34 (%)	1.35 ± 0.76	1.51 ± 1.12	0.343
KDR/CD34 (%)	1.26 ± 0.82	1.20 ± 0.73	0.623
Circulating level of EPCs at day 21			
CD31/CD34 (%)	1.64 ± 0.80	2.30 ± 1.53	0.04
KDR/CD34 (%)	1.23 ± 0.73	1.78 ± 1.26	0.004
RBC count (× 10^6^/ml) on day 21	4.49 ± 0.72	4.47 ± 0.70	0.839
Hemoglobin (g/dl) on day 21	13.39 ± 1.73	13.52 ± 1.85	0.693
Hematocrit (%) on day 21	40.12 ± 4.88	40.54 ± 5.22	0.661
WBC count (× 10^3^/ml) on day 21	7.36 ± 2.41	7.09 ± 3.14	0.272

Data are expressed as mean ± standard deviation of patients. Group 1, without erythropoietin treatment; Group 2, with erythropoietin treatment. EPC, endothelial progenitor cell; RBC, red blood cell; WBC, white blood cell.

### Comparison of short-term and long-term neurological status and long-term clinical outcome between groups 1 and 2

The scores from the NIHSS and the Barthel Index upon presentation (at 48 hours after acute IS) were similar between groups 1 and 2 (see Table 3). Additionally, the short-term (≤90-day) mortality and the Barthel Index on day 90 did not differ between groups 1 and 2. Furthermore, the mean long-term Barthel Index also did not differ between groups 1 and 2. Subgroup analysis, however, revealed that the occurrence of a long-term Barthel Index <35 or <40 and short-term (≤90-day) recurrent stroke were significantly lower in group 2 than in group 1. The incidences of recurrent stroke, major cardiac events, long-term recurrent stroke, and cumulative long-term mortality showed no differences between groups 1 and 2. The incidence of long-term MANE was significantly lower in group 2 than in group 1.

**Table 3:** **Comparison of short-term and long-term neurological status and long-term clinical outcome between the two groups of patients**

**Variable**	**Group 1 (** ***n*** **= 71)**	**Group 2 (** ***n*** **= 71)**	***P*** **value**
NIHSS at 48 hours	7.8 ± 4.5	7.2 ± 4.6	0.606
Barthel Index at 48 hours	55.4 ± 35.2	52.9 ± 29.4	0.631
Short-term (≤90-day) mortality	2.8% (2)	1.4% (1)	0.560
Short-term (≤90-day) recurrent stroke	9.9% (7)	0% (0)	0.007
Barthel Index on day 90	67.1 ± 34.5	70.5 ± 36.2	0.631
Long-term Barthel Index	72.1 ± 40.9	79.2 ± 33.3	0.332
Long-term Barthel Index <40	25.8% (15/58)	8.2% (5/61)	0.021
Long-term Barthel Index <35	29.3% (17/58)	10.3% (6/61)	0.007
Long-term recurrent stroke	23.9% (17)	15.5% (13)	0.206
Major adverse cardiac events^a^	9.9% (7)	5.6% (4)	0.346
Accumulative long-term mortality	18.3% (13)	14.1% (10)	0.494
Long-term MANE	49.3% (35)	32.4% (23)	0.04

Data are expressed as mean ± standard deviation or percentage (number) of patients. Group 1, without erythropoietin treatment; Group 2, with erythropoietin treatment. MANE, major adverse neurological event (defined as, long-term Barthel Index <35, recurrent stroke, or death after day-90 ischemic stroke); NIHSS, National Institutes of Health Stroke Scale. ^a^Defined as myocardial infarction or death.

### Univariate and multivariate logistic regression analysis of predictors for long-term Barthel Index <35

Univariate analysis (Table 4) of baseline variables in Tables 1, 2 and 3 showed that a history of previous stroke was significantly predictive of a long-term severe neurological deficit (that is, Barthel Index <35). Conversely, EPO therapy, an increased 21-day circulating EPC level (CD31CD34^+^), and angiotensin-converting enzyme inhibitor/angiotensin II type I receptor blocker therapy were significantly and strongly associated with freedom from long-term severe neurological deficit. Multivariate analysis (Table 5) showed that a history of previous stroke was a significant independent predictor of long-term severe neurological deficit, whereas EPO therapy was significantly and independently predictive of freedom from severe neurological deficit.

**Table 4:** **Univariate logistic regression analysis of predictors for long-term Barthel Index <35 after ischemic stroke** ^**a**^

**Variables**	**Odds ratio**	**95% confidence interval**	***P*** **value**
ACEI/ARB	0.26	0.001 to 0.573	0.021
Old stroke history	15.066	1.180 to 192.33	0.037
CD31/CD34 level at day 21	0.372	0.144 to 0.957	0.04
Erythropoietin	0.216	0.052 to 0.879	0.035

ACEI/ARB, angiotensin-converting enzyme inhibitor/angiotensin II type I receptor blocker. ^a^Long-term Barthel Index defined as the measurement that represented the final neurological status estimated at the end of the study period.

**Table 5:** **Multiple stepwise logistic regression analysis of predictors for long-term Barthel Index <35 after ischemic stroke**

**Variable**	**Odds ratio**	**95% confidence interval**	***P*** **value**
Old stroke history	4.600	1.266 to 16.715	0.020
Erythropoietin	0.235	0.064 to 0.863	0.029

### Univariate and multiple stepwise Cox regression analysis of predictors for long-term MANE after ischemic stroke

Univariate Cox regression analysis (Table 6) showed that increased age, previous stroke, and increased creatinine level were significantly predictive of long-term MANE. In contrast, circuiting levels of EPCs, EPO, and Barthel Index on day 90 therapy were three significant predictors of freedom from long-term MANE. Multivariate Cox regression analysis (Table 7) showed that increased age and creatinine level were significantly and independently predictive of long-term MANE, whereas EPO therapy and higher Barthel Index on day 90 were the only significant independent predictors of freedom from MANE.

**Table 6:** **Univariate Cox regression analysis of predictors for MANE after ischemia stroke**

**Variable**	**Hazards ratio**	**95% confidence interval**	***P*** **value**
Age (years)	1.032	1.007 to 1.057	0.012
Old stroke by history	1.890	1.091 to 3.274	0.023
Creatinine level (mg/dl)	1.834	1.12 to 3.026	0.016
CD31/CD34 (%)	0.740	0.585 to 0.981	0.036
Erythropoietin	0.568	0.335 to 0.961	0.024
Barthel Index on day 90	0.988	0.980 to 0.996	0.004

MANE, major adverse neurological event.

**Table 7:** **Multiple stepwise Cox regression analysis of predictors for MANE after ischemia stroke**

**Variable**	**Hazards ratio**	**95% confidence interval**	***P*** **value**
Age (years)	1.020	1.009 to 1.031	0.037
Creatinine	2.125	1.214 to 3.717	0.008
Erythropoietin	0.564	0.330 to 0.965	0.037
Barthel Index on day 90	0.990	0.981 to 0.999	0.024

MANE, major adverse neurological event.

### Correlation between long-term Barthel Index <35 and clinical outcome

**Figure 1:**
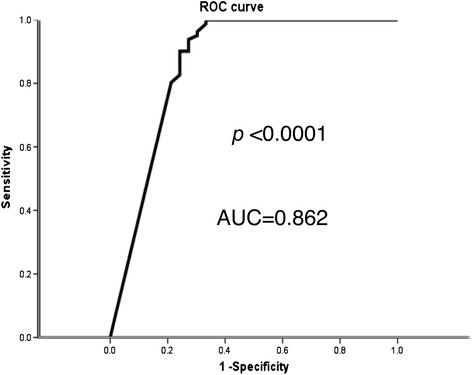
**Correlation between long-term Barthel Index <35 and clinical outcome.** Receiver operating characteristics (ROC) curve analysis revealed long-term Barthel Index <35 was the most powerful predictor of long-term major adverse neurological event with a sensitivity of 93.8% and a specificity of 72.7%, *P* <0.001. AUC, area under the curve.

Although the tabulated data (Tables 2 and 3) and the receiver operating characteristics curve analysis (Figure 1) each demonstrated a significant difference between the control and the EPO-treated cohorts using a Barthel Index cutoff point for severe outcome of either <40 or <35, the cutoff value of <35 proved to be the most powerful predictor of long-term MANE with a sensitivity of 93.8% and a specificity of 72.7% (that is, *P* <0.0001, area under the curve = 0.862).

### Kaplan–Meier survival plot for determining the long-term MANE-free rate

**Figure 2:**
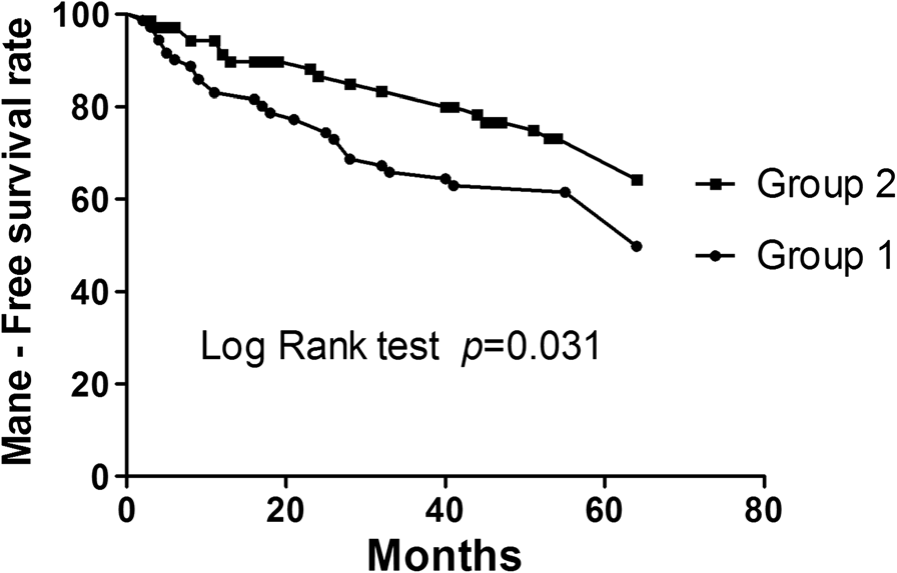
**Determining the long-term major adverse neurological event-free rate.** Kaplan–Meier survival plot. Five-year cumulative (log-rank test) freedom from major adverse neurological event (MANE) was significantly higher in group 2 than in group 1 patients, *P* = 0.031.

The log-rank test demonstrated that long-term freedom from MANE was significantly higher in group 2 than in group 1 (Figure 2) (*P* = 0.031).

## Discussion

This study, which investigated the impact of EPO therapy on long-term clinical outcome for IS patients, yielded several striking findings. First, long-term severe neurological deficit (that is, Barthel Index <35) and long-term MANE were remarkably lower in patients with EPO therapy than those with placebo. Second, EPO therapy and higher Barthel Index on day 90 were strongly and independently predictive of freedom from long-term severe neurological deficit and long-term MANE. Our findings therefore highlight that EPO could be considered as an alternative therapeutic option for acute IS patients who are not candidates for tPA.

Numerous clinical trials have previously extensively investigated the clinical outcomes in patients after acute IS [3,4,6,26,32-34]. Those IS patients who had higher NIHSS usually had poor prognostic outcomes after acute IS [23,26,33]. Thus, many investigators tried to find new modalities other than the traditional therapy for IS patients. Of these new modalities, thrombolysis with tPA is the most effective therapy for improving the prognostic outcome [9-11]. However, relatively higher intracranial bleeding that would cause high in-hospital mortality was a concern. Of importance was the fact that not all acute IS patients fit the criteria for tPA therapy [9-11]. EPO therapy has therefore been extensively investigated as it could be an alternative option for those patients not suitable for thrombolytic therapy [6,24,35-37]. Although not all of the clinical trial results of EPO therapy are consistent, the results of our study [24] and previous studies [16,38] have revealed that EPO therapy significantly improved clinical outcomes in patients after acute IS. Surprisingly, no long-term data have been reported by those previous clinical trials. Importantly, this is the first report of long-term follow-up (that is, up to 5 years) in a prospective, randomized, and placebo-controlled trial of EPO therapy for patients who were not suitable for tPA therapy.

One negative but important finding was that the incidence of recurrent stroke during long-term follow-up and long-term mortality did not differ between the two groups. Additionally, the mean long-term Barthel Index was similar between the two groups. Subgroup analysis, however, showed that severe long-term neurological deficit (that is, long-term Barthel Index <35) was substantially lower in patients who had received EPO therapy than in the placebo-control group. Furthermore, the incidence of long-term MANE was significantly lower in the EPO-treated group than in the placebo-control group. Of particular importance, EPO therapy and higher Barthel Index at day 90 were significantly and independently predictive of freedom from long-term severe neurological deficit and long-term MANE. It is well known that a severe neurological deficit after acute IS increases the social burden, detrimentally affects the patient’s quality of life and social reintegration [39-41], and is strongly associated with unfavorable long-term clinical outcomes. Reflecting upon these factors [7,8,23,24,39-41], our findings highlight that EPO should be considered an alternative therapeutic modality for patients who were not suitable for tPA therapy.

The underlying mechanisms of EPO therapy for reducing long-term severe neurological deficit and MANE remain uncertain. Interestingly, our previous study [24] showed that EPO therapy enhanced circulating numbers of EPCs [24], which, in turn, participated in the repair of cerebral vasculature. Additionally, circulating EPCs contributed to the maintenance and repair of cerebral vasculature in ischemia-related cerebrovascular dysfunction [42]. In the present study, we found that the level of circulating EPCs (Tables 4 and 5) by day 21 after IS was significantly increased in patients with EPO treatment compared with placebo-control patients. Accordingly, we suggest that the increased numbers of circulating EPCs might be recruited into the ischemic region to augment local angiogenesis and vasculogenesis, with resultant increased cerebral blood flow and improved neurological function. These findings suggest that EPO therapy improving neurological dysfunction may occur through peripheral (that is, increased circulating EPC level and then mobilization to the brain ischemic zone for angiogenesis) rather than central (directly protected neurons from apoptosis/death) effects. Additionally, improvement of anemia may be another possible effect on improving stroke outcome because most patients have a drop in hematocrit/red blood cells count after acute IS.

Increased age [43], previous stroke [23,24], and increased creatinine [43] are well recognized as important predictors of unfavorable clinical outcomes in the setting of cardiovascular and cerebrovascular diseases. In the present study, we found that a history of previous stroke was an independent predictor of long-term severe neurological dysfunction. Additionally, increased age and increased creatinine level were found to be independently predictive of long-term MANE. In this way, our findings were consistent with the findings of previous studies [23,24,43].

## Conclusion

Patients who received EPO therapy had significantly less long-term severe neurological deficit and significantly less long-term MANE compared with placebo-control patients. The results of our study encourage the use of EPO for patients with acute IS who are not candidates for tPA thrombolysis.

### Study limitations

This study has limitations. First, the sample size of this clinical trial was relatively small. Additionally, because of restrictive inclusion criteria, some critically ill patients were excluded during the initial enrollment period. The long-term mortality rate was thus relatively low in the current study. Accordingly, we cannot completely rule out a statistical distortion of significant differences of long-term mortality and recurrent stroke between these two groups of patients. Second, the incompleteness of follow-up data (due to patients withdrawn from the study or lost to follow-up) introduces uncertainty regarding interpretation of the differences in clinical outcome between these two groups of patients. Without measuring cerebrospinal fluid concentration of EPO, we did not know whether the improvement of neurological function was due to central or peripheral effect or due to both of them.

## Key messages

- EPO treatment significantly ameliorates long-term clinical outcomes in patients after acute IS.
- The results of the present study encourage the use of EPO for patients with acute IS who are not candidates for thrombolytic therapy.

## Abbreviations

EPC: endothelial progenitor cell
EPO: erythropoietin
IS: ischemic stroke
MANE: major adverse neurological event
MRI: magnetic resonance imaging
NIHSS: National Institutes of Health Stroke Scale
tPA: tissue plasminogen activator
