# Erratum to: Erythropoietin improves long-term neurological outcome in acute ischemic stroke patients: a randomized, prospective, placebo-controlled clinical trial

**DOI:** 10.1186/s13054-016-1256-y

**Published:** 2016-03-31

**Authors:** Tzu-Hsien Tsai, Cheng-Hsien Lu, Christopher Glenn Wallace, Wen-Neng Chang, Shu-Feng Chen, Chi-Ren Huang, Nai-Wen Tsai, Min-Yu Lan, Pei-Hsun Sung, Chu-Feng Liu, Hon-Kan Yip

**Affiliations:** Division of Cardiology, Department of Internal Medicine, Kaohsiung Chang Gung Memorial Hospital, Kaohsiung 123, Ta Pei Road, Niao Sung Hsiang, Kaohsiung, Hsien 83301 Taiwan; Department of Neurology, Kaohsiung Chang Gung Memorial Hospital, Kaohsiung 123, Ta Pei Road, Niao Sung Hsiang, Kaohsiung, Hsien 83301 Taiwan; Department of Plastic Surgery, University Hospital of South Manchester, Southmoor Road, Manchester, M23 9LT UK; Department of Emergency Medicine, Kaohsiung Chang Gung Memorial Hospital, Kaohsiung 123, Ta Pei Road, Niao Sung Hsiang, Kaohsiung, Hsien 83301 Taiwan; Center for Translational Research in Biomedical Sciences, Kaohsiung Chang Gung Memorial Hospital, Kaohsiung 123, Ta Pei Road, Niao Sung Hsiang, Kaohsiung, Hsien 83301 Taiwan; Institute of Shock Wave Medicine and Tissue Engineering, Kaohsiung Chang Gung Memorial Hospital, 123, Ta Pei Road, Niao Sung Hsiang, Kaohsiung, Hsien 83301 Taiwan; Chang Gung University College of Medicine, 123, Ta Pei Road, Niao Sung Hsiang, Kaohsiung, Hsien 83301 Taiwan

Unfortunately, the original version of this article [1] contained an error. The date of clinical trial registration for this study was 10^th^ January 2011, not 10^th^ October 2008 as reported in the original article.
